# *Nocardia aciditolerans* sp. nov., isolated from a spruce forest soil

**DOI:** 10.1007/s10482-013-9887-3

**Published:** 2013-02-01

**Authors:** Patrycja Golinska, Dylan Wang, Michael Goodfellow

**Affiliations:** 1 School of Biology, University of Newcastle, Newcastle upon Tyne, NE1 7RU UK; 2 Institute of Microbiology, Chinese Academy of Sciences, Beijing, 100101 People’s Republic of China; 3 Department of Microbiology, Nicolaus Copernicus University, 87 100 Torun, Poland

**Keywords:** *Nocardia aciditolerans*, Polyphasic taxonomy, Spruce forest soil, Actinomycetes

## Abstract

Actinomycetes growing on acidified starch-casein agar seeded with suspensions of litter and mineral soil from a spruce forest were provisionally assigned to the genus *Nocardia* based upon colonial properties. Representative isolates were found to grow optimally at pH 5.5, have chemotaxonomic and morphological features consistent with their assignment to the genus *Nocardia* and formed two closely related subclades in the *Nocardia* 16S rRNA gene tree. DNA:DNA relatedness assays showed that representatives of the subclades belong to a single genomic species. The isolates were distantly associated with their nearest phylogenetic neighbour, the type strain of *Nocardia kruczakiae*, and were distinguished readily from the latter based on phenotypic properties. On the basis of these data it is proposed that the isolates merit recognition as a new species, *Nocardia aciditolerans* sp. nov. The type strain is isolate CSCA68^T^ (=KACC 17155^T^ = NCIMB 14829^T^ = DSM 45801^T^).

**Electronic supplementary material:**

The online version of this article (doi:10.1007/s10482-013-9887-3) contains supplementary material, which is available to authorized users.

## Introduction

The genus *Nocardia* is a member of the family *Nocardiaceae* of the order *Corynebacteriales* within the class *Actinobacteria* (Goodfellow et al. 2012). The genus is well defined and presently encompasses 86 species with validly published names (http://www.bacteria.cict.fr/qr/nocardiae.html) which form a monophyletic clade within the evolutionary radiation occupied by genera classified in the order *Corynebacteriales* (Goodfellow and Jones 2012). The taxonomic status of most of these species is supported by an abundance of genotypic and phenotypic data (Goodfellow and Maldonado 2012) although it is evident that the genus is underspeciated (Wang et al. 1999; Maldonado et al. 2000; Roth et al. 2003). Many of the recently described *Nocardia* species were isolated from clinical material and considered to be opportunistic pathogens of humans and animals (Brown-Elliott et al. 2006; Goodfellow and Maldonado 2012). Saprophytic nocardiae have received less attention though they are widely distributed in natural habitats, notably soil, where they have a role in the turnover of organic matter (Orchard 1979, 1981). There is evidence that some species synthesize bioactive compounds of potential industrial value, as exemplified by *Nocardia iowensis* (Lamm et al. 2009).

During the characterisation of acidiphilic and aciditolerant actinomycetes isolated from a coniferous forest soil, a number of strains were found to have colonial properties typical of nocardiae. In the present study, the taxonomic status of representatives of these strains was determined by using a polyphasic approach. The resultant data show that the isolates belong to a new *Nocardia* species, for which we propose the name *Nocardia aciditolerans* sp. nov.

## Materials and methods

### Sampling site

Environmental samples were taken from the litter and mineral horizons of a pure stand of *Picea sitchensis* Carriere (Sitka spruce) at the southern end of Hamsterley Forest, County Durham, UK (National Grid Reference NZ 066292) in October, 2011. The trees were planted in 1929 with individual trees about 3 metres apart. The site was flat with no herbaceous or moss understory. The spruce needles formed a well defined horizon which was divided into three layers; the L layer consisted of about 4 cm of intact needles, the F layer of 4 cm of partially decomposed but recognisable needles and the H layer of a black-brown amorphous mass of decomposed needles, humic material and other organic matter, approximately 3.5 cm deep. The mean pH, moisture and organic matter contents of the three litter layers were 4.9, 4.0 and 3.8; 49, 68 and 32 % and 98, 95 and 93 %, respectively. The litter layers overlay the mineral soil which consisted of an A_1_ horizon (mean pH 3.9) about 7 cm thick, below which was the C horizon of parent millstone grit (mean pH 4.1); the moisture and organic matter contents of the horizons were 35 and 25 %, and 20 and 14 %, respectively.

### Organisms, maintenance and culture conditions

Acidiphilic and aciditolerant filamentous actinomycetes were sought from the litter and mineral horizons of the spruce forest soil using a standard dilution plate procedure (Goodfellow et al. 1967). Serial dilutions of the five environmental samples were spread over the surfaces of starch-casein medium (Kűster and Williams 1964) with agar (SCA) and gellan gum (GG) as gelling agents; the media were supplemented with cycloheximide and nystatin (each at 50 μg ml^−1^) and adjusted to pH 4.5 with 1 N HCl. Aliquots (100 μl) of each dilution prepared from the environmental suspensions were spread over the surfaces of the selective isolation plates which had been dried for 15 min prior to inoculation, as recommended by Vickers and Williams (1987). The inoculated plates, four per dilution, were incubated at 28 °C for 4 weeks. In addition to typical streptomycete-like colonies, 130 small compact colonies covered with white aerial hyphae were detected on isolation plates seeded with suspensions from the H, A_1_, and C layers. These *Nocardia*-like colonies were subcultured from starch-casein media prepared with each of the gelling agents and found to grow optimally on starch-casein agar at pH 5.5, but poorly at pH 4.5 and 7.0. Seventeen of the isolates were randomly selected for further study, namely strains HGG34, HGG71, HGG12n, HSCA8, and HSCA46 from the H layer; A1GG7, A1GG56, A1GG10n, A1SCA40, A1SCA48 and A1SCA1n from the A_1_ horizon; and CGG42, CGG62, CGG22n, CSCA51, CSCA58 and CSCA68^T^ from the C horizon. These strains were maintained as hyphal fragments in glycerol (20 %, v/v) at −80 °C and on glucose-yeast extract agar (GYEA; Gordon and Mihm 1962) slopes adjusted to pH 5.5.

Biomass for the molecular systematic and most of the chemosystematic studies was prepared by growing the representative strains in shake flasks of GYE broth (pH 5.5) at 150 revolutions per minute at 28 °C for 3 weeks. Cells were harvested by centrifugation and washed twice in distilled water; biomass for the chemotaxonomic analyses was freeze dried and that for the molecular systematic work stored at −20 °C. Biomass for the cellular fatty acid analysis carried out on isolate CSCA68^T^ was harvested from modified Bennett’s broth (Jones 1949), adjusted to pH 5.5, after incubation at 28 °C for 7 days.

### Phylogenetic analyses

Genomic DNA was extracted from the representative isolates using a GenElute™ Bacterial Genomic Kit (Sigma), according to the instructions of the manufacturer, albeit with lysozyme at 45 mg ml^−1^ and incubation overnight at 37 °C. The 16S rRNA genes were amplified using the universal primers p27f and p1525r (Lane 1991) under the following conditions: 1 μl DNA template (final concentration 100 ng ml^−1^), 5 μl 10× DNA polymerase buffer (Bioline), 3 μl MgCl_2_ (50 mM stock solution, Bioline), 1.6 μl of UTPs (12.5 mM stock mixture, Bioline), 1 μl of each primer (10 μM stock solution) and 1 μl polymerase (5 U, Bioline). The amplified products were separated by electrophoresis, purified with an ExoSap-IT kit (USB Corporation, Ohio, USA) and directly sequenced using a Dye Deoxy Terminator Cycle Sequencing Kit (Applied Biosystems), as described by Chun and Goodfellow (1995).

Nearly complete 16S rRNA gene sequences of the isolates (~1,400 nucleotides [nt]) were compared with corresponding sequences of the most closely related type strains using the EzTaxon server (Kim et al. 2012). Phylogenetic trees based on the aligned sequences were inferred using the maximum-likelihood (Felsenstein 1981) maximum parsimony (Fitch 1971) and neighbour-joining (Saitou and Nei 1987) tree-making algorithms drawn from the MEGA 5 (Tamura et al. 2011) and PHYML (Guindon and Gascuel 2003) software packages. An evolutionary distance matrix was generated using the distance model of Jukes and Cantor (1969). Confidence values of branches of the phylogenetic tree were determined in a bootstrap analysis based on 1,000 resampling of the neighbour-joining dataset (Felsenstein 1985) using the MEGA 5 software. The tree was rooted using the rRNA gene sequence of *Nocardia acidivorans* GW4-1778^T^ (accession number AM40 2972). Similarly, an expanded neighbour-joining tree was generated based on almost complete 16S rRNA gene sequences of the isolates and corresponding sequences of the type strains of *Nocardia* species, including those of the two acidotolerant taxa, *Nocardia jiangxiensis* and *Nocardia miyunensis* (Cui et al. 2005).

### Chemotaxonomy

Isolates HGG71, A1GG7, A1SCA48, CGG62, CGG22n, CSCA51 and CSCA68^T^ were examined for chemical markers known to be of value in nocardial systematics (Goodfellow and Maldonado 2012). Standard chromatographic procedures were used to determine the isomers of diaminopimelic acid (Staneck and Roberts 1974), mycolic acids (Minnikin et al. 1975) and whole-organism sugars (Hasegawa et al. 1983), using appropriate controls. Isolates CSCA68^T^ and A1SCA48 were examined for the presence of diagnostic menaquinones (Collins 1985) and polar lipids (Minnikin et al. 1984) with *Nocardia brasiliensis* strain NBRC 14402^T^ (ATCC 19296^T^) as control. Cellular fatty acids of isolate CSCA68^T^ were extracted, methylated and determined by gas chromatography (Hewlitt Packard instrument 6890) and analysed using the standard Sherlock Microbial Identification (MIDI) system, version 5 (Sasser 1990). The G+C mol% of the DNA of isolates A1SCA48 and CSCA68^T^ were determined following the procedure described by Gonzalez and Saiz-Jimenez (2002).

### DNA:DNA relatedness

The DNA:DNA relatedness value (∆Tm) between isolates CSCA68^T^ and A1SCA48 were determined using a fluorimetric method (Gonzalez and Saiz-Jimenez 2005), the optimal temperature for renaturation (Tm) was calculated using the equation *Tor* −0.51 (% GC) + 41. The melting temperature (Tm) at which 50 % of the initial double stranded DNA denatured into single-stranded DNA for isolate CSCA68^T^ and the isolates CSCA68^T^: A1SCA 48 hybrid DNA preparations was compared and the difference (∆Tm) calculated.

### Cultural, morphological and staining properties

All of the isolates were examined for cultural and morphological features following growth on standard media at 28 °C for 3 weeks. Cultural properties were investigated using tryptone-yeast extract, yeast extract-malt extract, oatmeal, inorganic-salts starch, glucose-asparagine, peptone-yeast extract-iron and tyrosine agars (International *Streptomyces* Project media 1–7 respectively; Shirling and Gottlieb 1966). Gram- and alcohol-acid fast staining were carried out following growth on glucose-yeast extract agar for 14 days at 28 °C using Hucker’s (Gerhardt 1981) and Ziehl-Neelsen (Gordon 1967) methods. The morphological properties of mycelia taken from GYEA plates were observed under a light microscope, after Gram staining.

### Phenotypic tests

All of the isolates were examined for a broad range of phenotypic properties known to be of value in nocardial systematics (Goodfellow 1971; Isik et al. 1999; Goodfellow and Maldonado 2012) with incubation at 28 °C for 3 weeks. The pH, temperature and sodium chloride tolerance tests were carried out using GYEA as the basal medium (Gordon and Mihm 1957).

## Results

### 16S rRNA gene sequencing and DNA:DNA relatedness studies

**Fig. 1:**
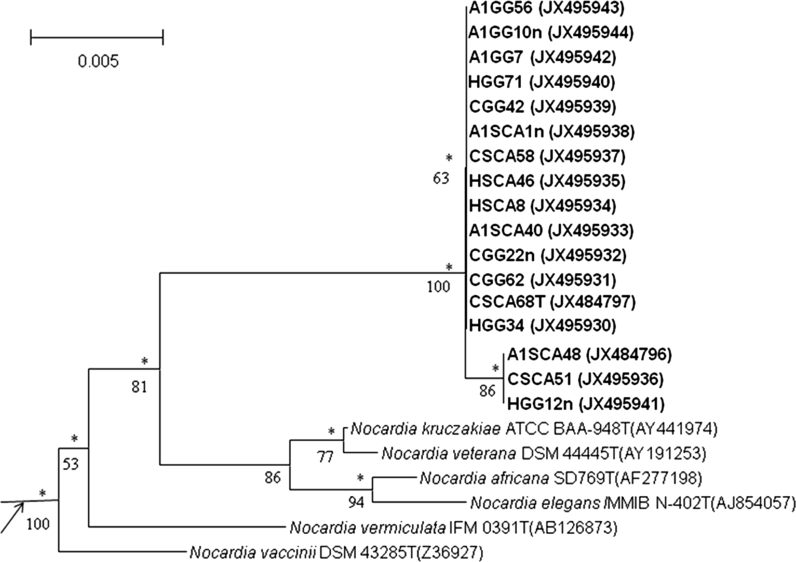
Neighbour-joining tree based on nearly complete 16S rRNA gene sequences showing relationships between the isolates and between them and the most closely related *Nocardia* species. Numbers at the nodes indicate the levels of bootstrap support based on a neighbour-joining analyses of 1,000 re-sampled datasets, only values above 50 % are given. *Asterisks* indicate the branches of the tree that were also recovered using the maximum-likelihood and maximum-parsimony tree-making algorithms. *T* type strain. *Bar* 0.005 substitutions per nucleotide position.The root position of the tree was obtained using *Nocardia acidivorans* GW4-1778^T^ as outgroup

Near complete 16S rRNA gene sequences for strains A1SCA48, CSCA68^T^ and HGG34^,^ CGG62^,^ CGG22n, A1SCA40, HSCA8, HSCA46, CSCA51, CSCA58, A1SCA1n, CGG42, HGG71, HGG12n, A1GG7, A1GG56, A1GG10n (GenBank accession numbers: JX484796–JX484797 and JX495930–JX495944, respectively) were determined. The sequences of all of 17 isolates formed a well-defined clade in the *Nocardia* 16S rRNA gene tree, a result underpinned by each of the tree-making algorithms and by a 100 % bootstrap value (Fig. 1). It is evident from the Figure that they formed two subclades, the larger of which included isolate CSCA68^T^. Members of the two subclades shared a 16S rRNA gene similarity of between 99.4 and 99.8 %, values equivalent to between 2 and 7 nt differences. The isolates were most closely related to the type strain of *Nocardia kruczakiae* sharing 16S rRNA gene similarities with the latter within the range 97.8–98.2 %, values that corresponded to between 25 and 30 nt differences at between 1,392 and 1,434 locations. The isolates were also distantly associated with the type strains of *Nocardia africana* (96.7–97.0 %), *Nocardia elegans* (97.4–97.7 % similarity) and *Nocardia veterana* (97.8–98.1 % similarity). It is apparent from the expanded neighbour-joining tree (Online supplementary Fig. 1) that the isolates are sharply separated from the type strains of *N. jiangxiensis* and *N. miyunensis* which form a clade supported by a 77 % bootstrap value. It is also apparent from this Figure that the clade encompassing the isolates is deep rooted but well within the *Nocardia* tree though the isolates are not closely related to any of the validly named *Nocardia* species.

The ∆Tm between isolate CSCA68^T^ g DNA and isolates CSCA68^T^: A1SCA48 hybrid DNA was 2 °C, a result that corresponds to a DNA:DNA similarity value of 80 % (Gonzalez and Saiz-Jimenez 2005), a value well above the 70 % cut off point recommended for assigning strains to the same genomic species (Wayne et al. 1987).

### Chemotaxonomic, cultural, morphological and phenotypic characteristics

**Fig. 2:**
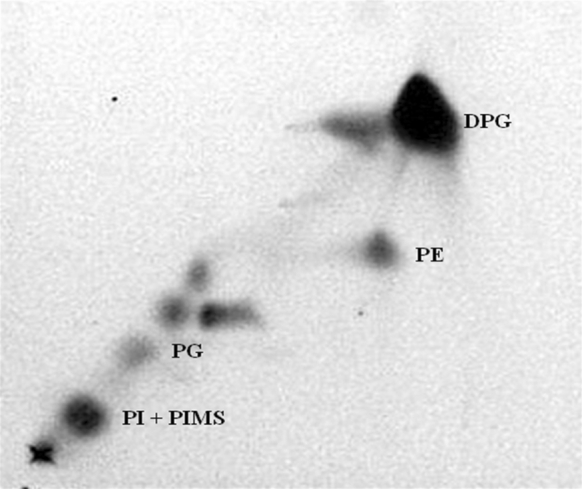
Two-dimensional thin-layer chromatography of polar lipids of isolate CSCA68^T^ stained with molybdenum blue spray (Sigma). Chloroform: methanol: water (32.5: 12.5: 2.0) was used in the first direction and chloroform: acetic acid: methanol: water (40: 7.5: 6: 2) in the second direction. *DPG* diphosphatidylglycerol, *PE* phosphatidylethanolamine, *PI* phosphatidylinositol, *PG* phosphatidylglycerol and *PIMS* phosphatidylinositol mannosides

Whole-organism hydrolysates of seven representative isolates were rich in *meso*-diaminopimelic acid (*meso*-A_2_pm), contained major amounts of arabinose and galactose with a trace of ribose and mycolic acids that co-chromatographed with those of *N. brasiliensis* NBRC 14402^T^ (ATCC 19296^T^). Isolates CSCA68^T^ and A1SCA48 were found to possess hexahydrogenated menaquinones with eight isoprene units where the last two isoprene units were cyclized (MK8 [H_4_ cyclo]) as the predominant isoprenologue. The strains were determined to contain diphosphatidylglycerol (DPG); phosphatidylethanolamine (PE), phosphatidylinositol (PI) and phosphatidylinositol mannosides (PIMS) as major polar lipids and a trace of phosphatidylglycerol (PG), as exemplified in Fig. 2. The major cellular fatty acids of isolate CSCA68^T^ were identified as C_16:0_ (46.3 %), C_18:1_ ω9c (13.5 %) and 10-methyl C_18:0_ (tuberculostearic acid; 11.8 %). The genomic DNA G + C contents of isolates A1SCA48 and CSCA68^T^ were determined to be the same, namely 71.3 mol%.

All of the isolates were observed to form an extensively branched substrate mycelium which carried light gray aerial hyphae on ISP7 agar following incubation at 28 °C for 3 weeks. The isolates were found to grow well on glycerol-asparagine, tyrosine, tryptone-yeast extract and yeast extract-malt extract agars (ISP media 5, 7, 1 and 2, respectively), poorly on oatmeal agar (ISP medium 3) and either poorly or not at all on peptone-yeast extract-iron agar (ISP medium 6) and inorganic salt starch agar (ISP medium 4). Sparse to moderate amounts of white to gray aerial hyphae were observed following growth on glycerol-asparagine and tyrosine agars. Isolates A1GG7, A1GG10n, CSCA68^T^ and HSCA46 were observed to produce brown to orange pigments on tyrosine agar. It can be seen from Table 1 that the isolates share many phenotypic traits; grow on a broad range of compounds as sole carbon sources, are relatively inactive in the biochemical and degradative tests, and do not grow at 40 °C, at either pH 4.0 or 8.0 or in the presence of 3 % w/v sodium chloride.

**Table 1:** Phenotypic properties of the aciditolerant isolates

Characteristics	A1SCA48	CSCA51	CGG62	HGG71	A1SCA1n	HSCA8	CSCA68^T^	HGG34	A1SCA40
Sole carbon sources 1 % (w/v)									
Adonitol	–	+	–	–	–	+	–	–	–
Amygdalin	–	–	+	–	–	–	+	+	–
l-Arabinose	–	+	–	–	–	+	+	–	+
d-Fructose	–	++	++	–	–	++	++	++	–
Glycogen	–	–	+	–	–	–	–	–	–
Inulin	–	–	+	–	–	+	+	+	–
d-Raffinose	–	+	+	–	–	–	+	+	–
d-Sorbitol	–	+	–	–	–	+	+	–	–
d-Trehalose	–	++	++	–	++	++	++	++	–
Xylitol	–	–	–	–	–	+	+	–	–
Sole carbon sources 0.1 % (v/w)									
Sodium succinate	–	+	+	+	–	+	+	+	+
Sole carbon & nitrogen sources									
Acetamide	+	+	+	–	–	+	+	+	+
l-Alanine	–	++	++	++	–	++	++	++	++
l-Asparagine	–	+++	+++	+++	+++	+++	+++	+++	–
l-Phenylalanine	++	++	–	–	++	++	–	–	–
Uric acid	+	+	–	–	–	–	–	–	–

Characteristics	CSCA58	CGG22n	HSCA46	CGG42	HGG12n	A1GG56	A1GG10n	A1GG7
Sole carbon sources 1 % (w/v)								
Adonitol	–	–	–	+	+	–	–	–
Amygdalin	–	–	+	+	+	+	–	–
l-Arabinose	–	–	–	+	–	–	–	–
d-Fructose	–	++	++	++	++	++	–	++
Glycogen	–	+	–	+	+	–	–	–
Inulin	–	–	+	+	+	+	–	–
d-Raffinose	–	–	+	+	–	–	–	–
d-Sorbitol	–	–	–	+	–	–	–	–
d-Trehalose	–	–	++	++	++	++	–	++
Xylitol	–	–	–	–	–	–	–	–
Sole carbon sources 0.1 % (v/w)								
Sodium succinate	+	+	+	+	+	+	+	+
Sole carbon & nitrogen sources								
Acetamide	–	–	+	+	+	–	+	+
l-Alanine	++	++	++	++	++	++	++	++
l-Asparagine	–	+++	+++	+++	+++	–	–	–
l-Phenylalanine	–	–	–	++	++	–	++	–
Uric acid	+	–	–	+	+	+	+	+

All isolates were positive for: nitrate reduction; hydrogen sulphide production; degradation of Tween 60; growth on *meso*-erythritol, d-galactose, d-glucose, *meso*-inositol, d-mannitol, d-mannose, d-melezitose, d-melibiose, d-ribose and d-xylose (1 %, w/v) and sodium fumarate (0.1 %, w/v) as sole carbon sources; growth on l-proline, l-serine and l-valine (0.1 %, w/v) as sole carbon and nitrogen sources, and at 10–37 °C, pH 5–7 and in the presence of 1 % w/v sodium chloride

All isolates were negative for: aesculin, allantoin and arbutin hydrolysis; degradation of adenine, casein, elastin, guanine, hypoxanthine, Tweens 20, 40 and 80, l-tyrosine, uric acid, xanthine and xylan; growth on d-cellobiose, dulcitol, d-lactose, d-maltose, l-rhamnose, d-salicin and d-sucrose (1 %, w/v) and ethanol, *iso*-amyl alcohol and 1,2—propanediol and *n*-propanol (1 %, v/v), and acetate, adipate, benzoate, butyrate, citrate, malonate, oxalate and l- (+)-tartrate (sodium salts at 0.1 %, w/v) as sole carbon sources, l-aspartic acid, ethanolamine, gelatin, l-glutamic acid and urea as sole carbon and nitrogen sources (0.1 %, w/v) and growth at 40 °C, pH 4 and 8, and in the presence of 3 %, w/v sodium chloride

Variable results were shown for: adonitol, amygdalin, l-arabinose, d-fructose, glycogen, inulin, d-raffinose, d-sorbitol, d-trehalose and xylitol (1 %, w/v) and sodium succinate (0.1 %, w/v) as sole carbon sources and acetmide, d-alanine, l-asparagine, l-phenylalanine and uric acid as sole carbon and nitrogen sources (0.1 %, w/v)

+ positive, − negative

A range of phenotypic properties can be weighted to distinguish the isolates from their nearest phylogenetic neighbours, including the type strain of *N. kruczakiae* (Table 2).

**Table 2:** Phenotypic properties that distinguish the 17 representatives isolates from the type strains of their closest phylogenetic neighbours

Characteristic	Isolates	*N. africana* DSM 44491^T^	*N. elegans* DSM 44890^T^	*N. kruczakiae* DSM 44887^T^	*N. veterana* DSM 44445^T^
Aesculin hydrolysis	–	–	+	+	–
Arbutin hydrolysis	–	–	–	+	+
Casein degradation	–	+	–	–	–
Growth on sole carbon sources (1 %, w/v)					
*meso*-Erythritol	+	–	–	–	–
d-Galactose	+	–	–	–	–
*meso*-Inositol	+	–	–	–	+
d-Maltose	–	–	–	+	–
d-Mannitol	+	–	–	–	–
l-Rhamnose	+	–	–	–	+
d-Xylose	+	–	–	–	–
Growth at:					
10 °C	+	–	–	–	–
45 °C	–	+	+	+	+

All of the strains grew on d-glucose as a sole carbon source (1 %, w/v) but were unable to degrade adenine, hypoxanthine, tyrosine or xanthine or to grow on d-cellobiose, d-sucrose (each at 1 %, w/v) or sodium acetate (0.1 %, w/v) as sole carbon sources

Data on the type strains were taken from Goodfellow and Maldonado (2012)

+ positive, − negative

## Discussion

All of the representative aciditolerant strains isolated from the litter and mineral horizons of the spruce forest soil at Hamsterley Forest gave chemotaxonomic and morphological characteristics of the genus *Nocardia* (Goodfellow and Maldonado 2012). They were observed to form an extensively branched substrate mycelium that fragmented into rod-like elements and produced whole-cell hydrolysates rich in *meso*-A_2_pm, arabinose and galactose (wall chemotype IV sensu Lechevalier and Lechevalier 1970). Isolate CSCA68^T^ was found to contain major proportions of straight-chain, saturated, unsaturated and tuberculostearic fatty acids, MK-8 (H_4ω_-cyclo) as the predominant isoprenologue, major amounts of DPG, PE, PI and PIMS (phospholipid type 2 after Lechevalier et al. 1977), mycolic acids with the same Rf value as those of *N. brasiliensis* strain NBRC 14402^T^ (ATCC 19296^T^) and DNA G+C values within the range 64–72 mol%.

The aciditolerant isolates formed a distinct phyletic branch in the *Nocardia* 16S rRNA gene tree sharing low 16S rRNA gene similarities with their closest neighbours, results consistent with their assignment to a separate species (Goodfellow and Maldonado 2012). Their phylogenetic relatives include their closest phylogenetic neighbour, the type strain of *N. kruczakiae*, a respiratory pathogen originally isolated from an immunocompromised patient (Conville et al. 2004). The isolates shared even lower 16S rRNA gene similarities with *N. jiangxiensis* 43401^T^ (CGMCC 4.1905^T^) and *N. miyunensis* 117^T^ (CGMCC 4.1904^T*)*^, aciditolerant strains isolated from acidic soils on an acidified selective isolation medium (Cui et al. 2005). It seems likely from these results that aciditolerant nocardiae may form an integral part of actinobacterial communities in acidic soils. The isolates can also be distinguished from the type strains of *N. miyunensis* and *N. jiangxiensis* for, unlike the latter, they grow at 10 °C but not on d-cellobiose, d-lactose, d-maltose, l-rhamnose, d-sucrose or sodium citrate as sole carbon sources nor do they hydrolyse aesculin.

The DNA:DNA homology assay showed that the representatives of the two closely related 16S rRNA gene subclades, namely strains CSCA68^T^ and A1SCA48 belong to the same genomic species. The results of the phenotypic tests were in line with this finding as the isolates from each of the subclades shared a diverse range of phenotypic properties. All of the representatives isolates can be separated from their nearest phylogenetic neighbours, the type strains of *N. africana*, *N. elegans*, *N. kruczakiae* and *N. veterana* using a combination of phenotypic tests found to be of value in nocardial systematics (Goodfellow and Maldonado 2012). Thus, the isolates, unlike *N. kruczakiae* DSM 44887^T^, grew at 10 °C, but not at 45 °C, and used *meso*-erythritol, d-galactose, *meso*-inositol, d-mannitol, l-rhamnose and d-xylose as sole carbon sources but were unable to hydrolyse aesculin or arbutin. It is interesting that the four type strains, all of which were isolated from clinical material, grew well at 45 °C (Conville et al. 2004).

It can be concluded from the genotypic and phenotypic data that the isolates form a distinct taxon that can be distinguished from all validly named *Nocardia* species, including *N. jiangxiensis* and *N. miyunensis*, the only aciditolerant *Nocardia* species validly described to date. The isolates are therefore considered to represent a novel *Nocardia* species, for which the name proposed is *N. aciditolerans*.

### Description of *N. aciditolerans* sp. nov.

*Nocardia aciditolerans* N.L. n. *acidum* (from L. adj. *acidus*, sour; L. pres. part. *tolerans*; N.L. past. adj. *aciditolerans*, acid tolerating).

Aerobic, Gram-positive, acid-alcohol-fast, aciditolerant actinomycetes that form an extensively branched, grayish yellow substrate mycelium which fragments into irregular rod- and coccoid-like elements after 14 days at 28 °C on glucose-yeast extract agar. Forms an extensively branched substrate mycelium bearing white or light gray aerial hyphae on glycerol—asparagine and tyrosine agars, respectively. Good growth also occurs on acidified tryptone-yeast extract and yeast extract-malt extract agars. Grows from pH 4.5–7.0 (optimally at pH 5.5), from 10 to 30 °C (optimally at 28 °C) and in the presence of 1 % but not 3 % w/v sodium chloride. Additional phenotypic properties are cited in the text or in Table 1. The cellular fatty acid profile of the type strain contains major proportions of C_16:0_, C_18:1_ ω9c, 10-methyl C_18:0_; lower proportions of C_14:0_, C_16:1_ ω9c, C_17:1_ ω8c, C_17:1_ ω5c, C_17:0_ and methyl C_17:0_, and traces of C_12:0_, C_13:0_, C_15:1_ ω8c, C_15:1_ ω5c, C_15:0_ 30H, C_18:3_ ω6c and C_18:0_. The DNA G+C content of the type strain is 71.3 mol%.

The type strain, CSCA 68^T^ (=KACC 17155^T^ = NCIMB 14829^T^ = DSM 45801^T^), was isolated from the C mineral horizon at Hamsterley Forest, County Durham, UK.

## Electronic supplementary material

Below is the link to the electronic supplementary material.

Supplementary Fig. 1 Neighbour-joining tree based on nearly complete 16S rRNA gene sequences (1292-1510 nt) showing relationships between the isolates and validly published *Nocardia* species. T, type strain. Bar, 0.005 substitutions per nucleotide position (PDF 25 kb)
