# Supplementary figures and images for: Nocardia aciditolerans sp. nov., isolated from a spruce forest soil
Source: Antonie Van Leeuwenhoek. 2013 Feb 1;103(5):1079–88. doi: 10.1007/s10482-013-9887-3 (PMC3621993; doi:10.1007/s10482-013-9887-3)

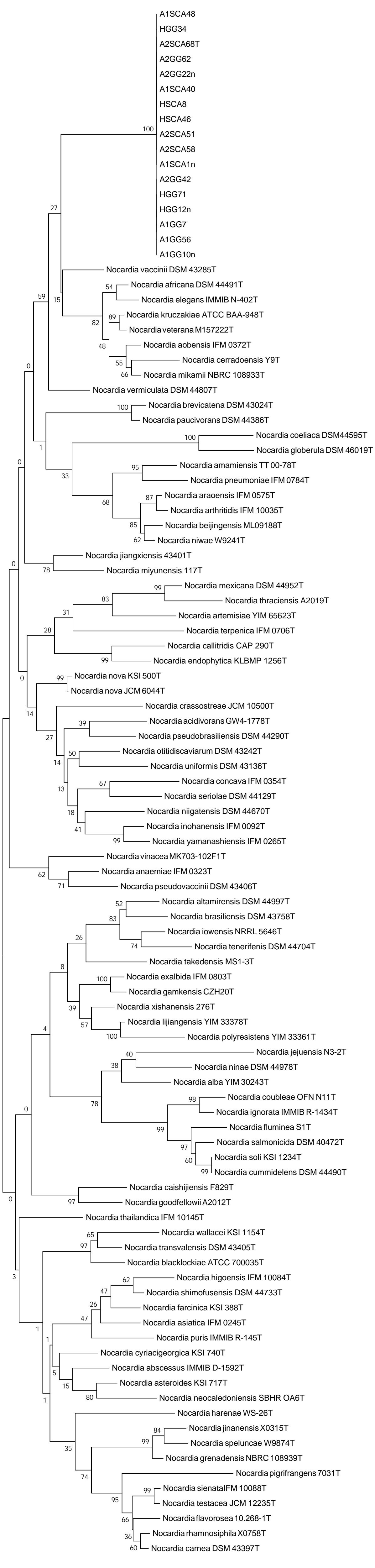

Supplement: Supplementary file 1 — Supplementary Fig. 1 Neighbour-joining tree based on nearly complete 16S rRNA gene sequences (1292-1510 nt) showing relationships between the isolates and validly published Nocardia species. T, type strain. Bar, 0.005 substitutions per nucleotide position (PDF 25 kb) [file 10482_2013_9887_MOESM1_ESM.pdf]
